# Impact of the COVID-19 Pandemic on the Use of Quaternary Ammonium Compound Disinfectants in Healthcare Facilities Within Selected States in the United States of America

**DOI:** 10.7759/cureus.75382

**Published:** 2024-12-09

**Authors:** Callie Moran, Kathrine Gaiko, Tillman Landers, Hannah L Paros, Chang Xu, Elizabeth Fernandez, Brooke Burwell, Tyler Steve, Sasha Margob, Marisela Davis, Karen Price, Madeleine E Chew, Sara Worrill, Brianna R Childress, Sara Safford, Janvi Patel, Jessica Marino, Ashley Azmoodeh, Theresa J McCann, Terry Hrubec

**Affiliations:** 1 Epidemiology and Public Health, Edward Via College of Osteopathic Medicine - Virginia, Blacksburg, USA; 2 Epidemiology and Public Health, Edward Via College of Osteopathic Medicine - Auburn, Auburn, USA; 3 Epidemiology and Public Health, Edward Via College of Osteopathic Medicine - Carolinas, Spartanburg, USA; 4 Preventive Medicine, Epidemiology, Biostatistics, Public Health, Edward Via College of Osteopathic Medicine - Virginia, Blacksburg, USA; 5 Anatomy/Embryology, Edward Via College of Osteopathic Medicine - Virginia, Blacksburg, USA

**Keywords:** covid-19, disinfectants, environmental exposures, public and environmental health, qacs, quaternary ammonium compounds, sars-cov-2

## Abstract

Introduction

Quaternary ammonium compounds (QACs) are the active ingredient in the majority of disinfectants approved for use against severe acute respiratory syndrome coronavirus 2 (SARS-CoV-2), the causative agent of the coronavirus disease 2019 (COVID-19). Although widely used, they have been linked to infertility and birth defects in animals, and have been shown to increase proinflammatory cytokines, decrease mitochondrial function, and disrupt sterol biosynthetic pathways in a dose-dependent manner in humans. This study examined if there was an increased use of QAC-based disinfectants among healthcare settings in response to the COVID-19 pandemic and aims to bring to light the negative health outcomes that this rise in QAC exposure may pose.

Methods

This hypothesis was explored using a telephone survey tool with both open-ended and closed-ended questions to assess changes in disinfection practices within hospitals, private practices, and dental practices. To ensure that the data were most representative of the United States, all states were ranked based on 28 health metrics tracked by the Kaiser Family Foundation. Those that ranked closest to the national average were Arizona, Florida, Illinois, Michigan, Pennsylvania, and Virginia. Healthcare facilities within these states were called at random and asked about changes in disinfection practices, specifically regarding the disinfectant type, product concentration, changes in the frequency of cleaning, and changes in the cleaning method. Additional data such as demographics, hospital ward type, and practice type were also obtained.

Results

QAC-based disinfectants were one of the most commonly used products in the surveyed hospitals, private practices, and dental practices both before and throughout the pandemic. Among all the medical facilities surveyed, approximately 80 to 90% indicated an increase in the frequency of cleaning. Within private practices, approximately 80 to 90% increased both spraying and wiping, while within dental practices, 75% increased wiping and only 60% increased spraying. Over 80% of hospitals reported an increase in all methods of disinfection within each ward type. The number of dental practices using QAC-containing disinfectants increased due to the pandemic. In hospitals, the class of disinfectant varied considerably by ward, but overall fewer of them used QAC-based disinfectants and more relied on peroxide-containing disinfectants.

Conclusions

This study identified increases in the disinfection frequency and changes to disinfection products within healthcare settings as a result of the COVID-19 pandemic. The predominant use of QAC-based products and the increased frequency of disinfection are important findings as they indicate heightened exposure. Emerging literature has identified adverse health outcomes in both animals and humans from contact with QACs. Increased disinfection during a pandemic or disease outbreak is critical to control the spread of disease; however, further research is needed to investigate whether the use of QAC-containing disinfectants to combat COVID-19 results in unintended disease burden in the healthcare workers.

## Introduction

Quaternary ammonium compounds (QACs) are one of the most common classes of chemicals found in disinfectants, detergents, pesticides, and personal care products [1]. The Environmental Protection Agency (EPA) has published a list of disinfectants effective against Severe Acute Respiratory Syndrome Coronavirus 2 (SARS-CoV-2) called “List N”, in which over half of the products contain QACs [2]. The products identified in the list are strictly for disinfecting hard surfaces, and inclusion in the list does not constitute an endorsement by the EPA. There was an expected increase in QAC use in response to the Coronavirus Disease 2019 (COVID-19) pandemic and perhaps even afterwards as a 10% increase per year is predicted in the worldwide utilization of disinfectants from 2020 to 2027 [3]. This class has long been considered highly efficacious, with one study indicating that the use of a QAC spray disinfectant significantly decreased the number of *Staphylococcal* and *Pseudomonas aeruginosa* bacteria and QAC wipes diminished bacteria by 99.9% overall [4]. These statistics are one reason why QAC-containing products are often the disinfectant of choice in healthcare settings.

QACs have long been considered safe with minimal toxicity risks, contributing to their frequent and widespread use in healthcare and household settings [4]. However, new data has begun to emerge over the last decade demonstrating the negative health effects associated with these chemicals. This is not unique to QACs as all biocides have inherent toxicological risks to animal and human health as well as to the environment [5]. In humans, QACs promote proinflammatory cytokines that can induce asthma and eczema through inhalation and contact [5]. They also increase systemic inflammation, decrease mitochondrial function, and alter sterol homeostasis in a dose-dependent manner [6]. In animal studies, these chemicals also cause birth defects and infertility [3,7,8]. These effects on human health are a direct juxtaposition to the presumed minimal toxicity and call into question the widespread use of QACs.

Moreover, these compounds are found in the environment with an estimated 75% of QACs used every year eventually entering the water through wastewater treatment systems [2]. Significant quantities have been identified in wastewater sludge due to their adsorption to particulates present in wastewater [9]. After sewage sludge is treated and dried, it is added to landfills, used as agricultural fertilizer or utilized in landscaping [10]. Although sanitation processes are in place, occasionally the chemicals are not filtered out. These will then accumulate in the soil to be taken up by crops and eventually enter the food supply [10]. This indicates that food and water are other sources of exposure to QACs for humans and animals, in addition to the direct contact with cleaning products mentioned above.

The objective of this paper is to identify whether disinfectant use, particularly those containing QACs, has increased across hospitals, private practices, and dental practices in response to the pandemic. Heightened use of QAC disinfectants is particularly concerning as it indicates an increased exposure to potentially harmful chemicals, especially in healthcare workers. By examining the association between the COVID-19 pandemic and QAC use, this study intends to contribute to the existing body of literature regarding human exposure to these chemicals and provide more insight into potential risks associated with them.

## Materials and methods

To test the hypothesis that QAC-containing disinfectant use has increased in medical settings during the COVID-19 pandemic, we developed and utilized a telephone survey tool with open-ended and closed-ended questions. The study assessed changes in disinfection practices and products within hospitals, private practices, and dental practices for two years from January 2021 to January 2023 within six states selected as representative of the country. Hospitals, private and dental practices were chosen as they represent different sectors of healthcare facilities and as such may have changed disinfection practices in ways unique to their role in providing healthcare. All facilities surveyed were identified and defined based on their inclusion in independently compiled national lists of medical facilities (see below for details). Their designation in this study as hospital, private practice, or dental facility was based on their inclusion and designation in the respective list.

Survey creation

To gather all-encompassing data, a telephone survey was developed in Qualtrix (Seattle, Washington, USA) to assess changes in disinfectant use due to the COVID-19 pandemic in a variety of healthcare settings including hospitals, private practices, and dental practices (Tables 1-3). The survey utilized a standardized script and question format with open-ended and closed-ended questions regarding disinfection practices. Survey validation was assessed following the guidelines given in Aithal and Aithal, 2020 [11]. Briefly, the questionnaire was developed and assessed for face and content validity through evaluation and review by experts and pilot test populations. Survey administrators were trained in the uniform administration of the survey and monitored for inter-administrator consistency. All survey questions comprised demographic information or facts about disinfection practices, coded in a structured, numerical format. No questions regarding personal opinions, attitudes, health-related information, intangible information like feelings, satisfaction, disinfectant efficacy etc. were asked.

The questions focused on COVID-19 related changes in disinfectant product, concentration, methods of cleaning (soaking, mopping, spraying, wiping), and the frequency of cleaning. Private practices and dental offices were also asked these questions regarding their facility as a whole and also within specific regions of the practice. Hospitals were asked to specify the details based on the types of wards present. The areas of interest within hospitals included inpatient wards, outpatient wards, intensive care units (ICU), operating theaters, immunocompromised wards, labor and delivery (L&D) units, and nurses’ stations. All facilities were asked to report their location in terms of urban, rural, or suburban, whether they were privately owned or by corporates, and their status as general or specialty clinics.

**Table 1:** Survey questionnaire for hospitals ICU: Intensive care unit; NICU: Neonatal intensive care unit; COVID: Coronavirus disease

SURVEY QUESTIONNAIRE FOR HOSPITALS								
1. Is the hospital privately or corporately owned?								
2. Is the hospital URBAN SUBURBAN or RURAL								
3. On average, how many patients are admitted yearly?								
4. How many beds does the hospital have?								
5. How many of these beds are for specialty care such as ICU, NICU, coronary, oncology, burn etc.?								
6. How many of these beds are general medical or surgical beds?								
7. Is there a specific person in the practice who makes the decisions for disinfection products and protocols? YES NO						If YES, what is the position title of this person?		
8. Is an outside service contracted to disinfect the outpatient wards? YES NO	If YES: Skip to question 9							
	If NO: Have the disinfection protocols in the outpatient wards changed because of COVID? YES NO		If NO: What class or classes of disinfectant are currently used? Jump to question 9					
			If YES: Has the type of disinfectant used in the outpatient wards changed because of COVID? YES NO			If NO: What class or classes of disinfectant are currently used?		
						If YES: What class or classes of disinfectant were used before the pandemic? What class or classes of disinfectant are used now?		
			Has the concentration of disinfectant used changed because of COVID? YES NO					
			Regarding the frequency of disinfection, is disinfecting occurring MORE OFTEN, LESS OFTEN, or ABOUT THE SAME as before COVID?					
			With regards to the outpatient wards, we would like to ask if there has been a change in the way disinfectants are applied. For each of the following application methods, can you indicate if it has INCREASED, DECREASED, or STAYED ABOUT THE SAME because of COVID?				Spray	
							Wipe	
							Mop	
							Soak	
							Other	
9. Is an outside service contracted to disinfect the inpatient wards? YES NO	If YES: Skip to question 10							
	If NO: Have the disinfection protocols in the inpatient wards changed because of COVID? YES NO		If NO: What class or classes of disinfectant are currently used? Jump to question 10					
			If YES: Has the type of disinfectant used in the inpatient wards changed because of COVID? YES NO			If NO: What class or classes of disinfectant are currently used?		
						If YES: What class or classes of disinfectant were used before the pandemic? What class or classes of disinfectant are used now?		
			Has the concentration of disinfectant used changed because of COVID? YES NO					
			Regarding the frequency of disinfection, is disinfecting occurring MORE OFTEN, LESS OFTEN, or ABOUT THE SAME as before COVID?					
			With regards to the inpatient wards, we would like to ask if there has been a change in the way disinfectants are applied. For each of the following application methods, can you indicate if it has INCREASED, DECREASED, or STAYED ABOUT THE SAME because of COVID?				Spray	
							Wipe	
							Mop	
							Soak	
							Other	
10. Is an outside service contracted to disinfect the ICU wards? YES NO	If YES: Skip to question 11							
	If NO: Have the disinfection protocols in the ICU changed because of COVID? YES NO		If NO: What class or classes of disinfectant are currently used? Jump to question 11					
			If YES: Has the type of disinfectant used in the ICU changed because of COVID? YES NO			If NO: What class or classes of disinfectant are currently used?		
						If YES: What class or classes of disinfectant were used before the pandemic? What class or classes of disinfectant are used now?		
			Has the concentration of disinfectant used changed because of COVID? YES NO					
			Regarding the frequency of disinfection, is disinfecting occurring MORE OFTEN, LESS OFTEN, or ABOUT THE SAME as before COVID?					
			With regards to the ICU, we would like to ask if there has been a change in the way disinfectants are applied. For each of the following application methods, can you indicate if it has INCREASED, DECREASED, or STAYED ABOUT THE SAME because of COVID?				Spray	
							Wipe	
							Mop	
							Soak	
							Other	
11. Is an outside service contracted to disinfect the operating theater? YES NO		If YES: Skip to question 12						
		If NO: Have the disinfection protocols in the operating theater changed because of COVID? YES NO		If NO: What class or classes of disinfectant are currently used? Jump to question 12				
				If YES: Has the type of disinfectant used in the operating theater changed because of COVID? YES NO	If NO: What class or classes of disinfectant are currently used?			
					If YES: What class or classes of disinfectant were used before the pandemic? What class or classes of disinfectant are used now?			
				Has the concentration of disinfectant used changed because of COVID? YES NO				
				Regarding the frequency of disinfection, is disinfecting occurring MORE OFTEN, LESS OFTEN, or ABOUT THE SAME as before COVID?				
				With regards to the operating theater, we would like to ask if there has been a change in the way disinfectants are applied. For each of the following application methods, can you indicate if it has INCREASED, DECREASED, or STAYED ABOUT THE SAME because of COVID?				Spray
								Wipe
								Mop
								Soak
								Other
12. Is an outside service contracted to disinfect the wards with immunocompromised patients? YES NO		If YES: Skip to question 13						
		If NO: Have the disinfection protocols in the wards with immunocompromised patients changed because of COVID? YES NO		If NO: What class or classes of disinfectant are currently used? Jump to question 13				
				If YES: Has the type of disinfectant used in the wards with immunocompromised patients changed because of COVID? YES NO	If NO: What class or classes of disinfectant are currently used?			
					If YES: What class or classes of disinfectant were used before the pandemic? What class or classes of disinfectant are used now?			
				Has the concentration of disinfectant used changed because of COVID? YES NO				
				Regarding the frequency of disinfection, is disinfecting occurring MORE OFTEN, LESS OFTEN, or ABOUT THE SAME as before COVID?				
				With regards to the wards with immunocompromised patients, we would like to ask if there has been a change in the way disinfectants are applied. For each of the following application methods, can you indicate if it has INCREASED, DECREASED, or STAYED ABOUT THE SAME because of COVID?				Spray
								Wipe
								Mop
								Soak
								Other
13. Is an outside service contracted to disinfect the labor / delivery wards? YES NO		If YES: Skip to question 14						
		If NO: Have the disinfection protocols in the labor / delivery wards changed because of COVID? YES NO		If NO: What class or classes of disinfectant are currently used? Jump to question 14				
				If YES: Has the type of disinfectant used in the labor / delivery wards changed because of COVID? YES NO	If NO: What class or classes of disinfectant are currently used?			
					If YES: What class or classes of disinfectant were used before the pandemic? What class or classes of disinfectant are used now?			
				Has the concentration of disinfectant used changed because of COVID? YES NO				
				Regarding the frequency of disinfection, is disinfecting occurring MORE OFTEN, LESS OFTEN, or ABOUT THE SAME as before COVID?				
				With regards to the labor / delivery wards, we would like to ask if there has been a change in the way disinfectants are applied. For each of the following application methods, can you indicate if it has INCREASED, DECREASED, or STAYED ABOUT THE SAME because of COVID?				Spray
								Wipe
								Mop
								Soak
								Other
14. Is an outside service contracted to disinfect the nurse’s stations? YES NO		If YES: Terminate survey						
		If NO: Have the disinfection protocols in the nurse’s stations changed because of COVID? YES NO		If NO: What class or classes of disinfectant are currently used? Terminate survey				
				If YES: Has the type of disinfectant used in the nurse’s stations changed because of COVID? YES NO	If NO: What class or classes of disinfectant are currently used?			
					If YES: What class or classes of disinfectant were used before the pandemic? What class or classes of disinfectant are used now?			
				Has the concentration of disinfectant used changed because of COVID? YES NO				
				Regarding the frequency of disinfection, is disinfecting occurring MORE OFTEN, LESS OFTEN, or ABOUT THE SAME as before COVID?				
				With regards to the nurse’s stations, we would like to ask if there has been a change in the way disinfectants are applied. For each of the following application methods, can you indicate if it has INCREASED, DECREASED, or STAYED ABOUT THE SAME because of COVID? Terminate survey				Spray
								Wipe
								Mop
								Soak
								Other

**Table 2:** Survey questionnaire for private practices

SURVEY QUESTIONNAIRE FOR PRIVATE PRACTICES					
1. Is the practice privately or corporately owned?					
2. What specialties does the practice include?					
3. Is the practice URBAN SUBURBAN or RURAL					
4. On average, how many patients are seen in a day?					
5. How many members are on the clinical staff (anyone outside of clerical/front desk staff)?					
6. How many patient rooms does the practice have?					
7. Is there a specific person in the practice who makes the decisions for disinfection products and protocols? YES NO			If YES, what is the position title of this person?		
8. Is an outside service contracted to disinfect the entire practice? YES NO	If NO: Skip to question 9				
9. Are the same disinfection products and protocols used throughout the entire practice? YES NO	If NO: Skip to question 10				
	If YES: Have the disinfection protocols in the practice changed because of COVID? YES NO	If NO: What class or classes of disinfectant are currently used?			
		If YES: Has the type of disinfectant used in the practice changed because of COVID? YES NO	If NO: What class or classes of disinfectant are currently used?		
			If YES: What class or classes of disinfectant were used before the pandemic? What class or classes of disinfectant are used now?		
		Has the concentration of disinfectant used changed because of COVID? YES NO			
		Regarding the frequency of disinfection, is disinfecting occurring MORE OFTEN, LESS OFTEN, or ABOUT THE SAME as before COVID?			
		With regards to the practice, we would like to ask if there has been a change in the way disinfectants are applied. For each of the following application methods, can you indicate if it has INCREASED, DECREASED, or STAYED ABOUT THE SAME because of COVID? Then terminate survey		Spray	
				Wipe	
				Mop	
				Soak	
				Other	
10. Is an outside service contracted to disinfect the patient rooms? YES NO	If YES: Skip to question 11				
	If NO: Have the disinfection protocols in the patient rooms changed because of COVID? YES NO	If NO: What class or classes of disinfectant are currently used? Jump to question 11			
		If YES: Has the type of disinfectant used in the patient rooms changed because of COVID? YES NO	If NO: What class or classes of disinfectant are currently used?		
			If YES: What class or classes of disinfectant were used before the pandemic? What class or classes of disinfectant are used now?		
		Has the concentration of disinfectant used changed because of COVID? YES NO			
		Regarding the frequency of disinfection, is disinfecting occurring MORE OFTEN, LESS OFTEN, or ABOUT THE SAME as before COVID?			
		With regards to the patient rooms, we would like to ask if there has been a change in the way disinfectants are applied. For each of the following application methods, can you indicate if it has INCREASED, DECREASED, or STAYED ABOUT THE SAME because of COVID?		Spray	
				Wipe	
				Mop	
				Soak	
				Other	
11. Is the waiting room open to patients? YES NO	If NO: Skip to question 12				
	If YES: Is an outside service contracted to disinfect the waiting room? YES NO	If YES: jump to question 12			
		If NO: Have the disinfection protocols in the waiting room changed because of COVID? YES NO	If NO: What class or classes of disinfectant are currently used? Jump to question 12		
			If YES: Has the type of disinfectant used in the waiting room changed because of COVID? YES NO	If NO: What class or classes of disinfectant are currently used?	
				If YES: What class or classes of disinfectant were used before the pandemic? What class or classes of disinfectant are used now?	
			Has the concentration of disinfectant used changed because of COVID? YES NO		
			Regarding the frequency of disinfection, is disinfecting occurring MORE OFTEN, LESS OFTEN, or ABOUT THE SAME as before COVID?		
			With regards to the waiting room, we would like to ask if there has been a change in the way disinfectants are applied. For each of the following application methods, can you indicate if it has INCREASED, DECREASED, or STAYED ABOUT THE SAME because of COVID?	Spray	
				Wipe	
				Mop	
				Soak	
				Other	
12. Is there an imaging room? YES NO	If NO: Skip to question 13				
	If YES: Is an outside service contracted to disinfect the imaging room? YES NO	If YES: jump to question 13			
		If NO: Have the disinfection protocols in the imaging room changed because of COVID? YES NO	If NO: What class or classes of disinfectant are currently used? Jump to question 13		
			If YES: Has the type of disinfectant used in the imaging room changed because of COVID? YES NO	If NO: What class or classes of disinfectant are currently used?	
				If YES: What class or classes of disinfectant were used before the pandemic? What class or classes of disinfectant are used now?	
			Has the concentration of disinfectant used changed because of COVID? YES NO		
			Regarding the frequency of disinfection, is disinfecting occurring MORE OFTEN, LESS OFTEN, or ABOUT THE SAME as before COVID?		
			With regards to the imaging room, we would like to ask if there has been a change in the way disinfectants are applied. For each of the following application methods, can you indicate if it has INCREASED, DECREASED, or STAYED ABOUT THE SAME because of COVID?	Spray	
				Wipe	
				Mop	
				Soak	
				Other	
13. Is there a sterilization room? YES NO	If NO: Skip to question 14				
	If YES: Is an outside service contracted to disinfect the sterilization room? YES NO	If YES: jump to question 14			
		If NO: Have the disinfection protocols in the sterilization room changed because of COVID? YES NO	If NO: What class or classes of disinfectant are currently used? Jump to question 14		
			If YES: Has the type of disinfectant used in the sterilization room changed because of COVID? YES NO	If NO: What class or classes of disinfectant are currently used?	
				If YES: What class or classes of disinfectant were used before the pandemic? What class or classes of disinfectant are used now?	
			Has the concentration of disinfectant used changed because of COVID? YES NO		
			Regarding the frequency of disinfection, is disinfecting occurring MORE OFTEN, LESS OFTEN, or ABOUT THE SAME as before COVID?		
			With regards to the sterilization room, we would like to ask if there has been a change in the way disinfectants are applied. For each of the following application methods, can you indicate if it has INCREASED, DECREASED, or STAYED ABOUT THE SAME because of COVID?	Spray	
				Wipe	
				Mop	
				Soak	
				Other	
14. Is there a laboratory area? YES NO	If NO: Terminate survey				
	If YES: Is an outside service contracted to disinfect the laboratory area? YES NO	If YES: Terminate survey			
		If NO: Have the disinfection protocols in the laboratory area changed because of COVID? YES NO	If NO: What class or classes of disinfectant are currently used? Terminate survey		
			If YES: Has the type of disinfectant used in the laboratory area changed because of COVID? YES NO	If NO: What class or classes of disinfectant are currently used?	
				If YES: What class or classes of disinfectant were used before the pandemic? What class or classes of disinfectant are used now?	
			Has the concentration of disinfectant used changed because of COVID? YES NO		
			Regarding the frequency of disinfection, is disinfecting occurring MORE OFTEN, LESS OFTEN, or ABOUT THE SAME as before COVID?		
			With regards to the laboratory area, we would like to ask if there has been a change in the way disinfectants are applied. For each of the following application methods, can you indicate if it has INCREASED, DECREASED, or STAYED ABOUT THE SAME because of COVID? Terminate survey	Spray	
				Wipe	
				Mop	
				Soak	
				Other	

**Table 3:** Survey questionnaire for dental practices

SURVEY QUESTIONNAIRE FOR DENTAL PRACTICES				
1. Is the practice privately or corporately owned?				
2. What specialties does the practice include?				
3. Is the practice URBAN SUBURBAN or RURAL				
4. On average, how many patients are seen in a day?				
5. How many members are on the clinical staff (anyone outside of clerical/front desk staff)?				
6. How many patient rooms does the practice have?				
7. Is there a specific person in the practice who makes the decisions for disinfection products and protocols? YES NO			If YES, what is the position title of this person?	
8. Is an outside service contracted to disinfect the entire practice? YES NO	If NO: Skip to question 9			
	If YES: Terminate survey			
9. Are the same disinfection products and protocols used throughout the entire practice? YES NO	If NO: Skip to question 10			
	If YES: Have the disinfection protocols in the practice changed because of COVID? YES NO	If NO: What class or classes of disinfectant are currently used?		
		If YES: Has the type of disinfectant used in the practice changed because of COVID? YES NO	If NO: What class or classes of disinfectant are currently used?	
			If YES: What class or classes of disinfectant were used before the pandemic? What class or classes of disinfectant are used now?	
		Has the concentration of disinfectant used changed because of COVID? YES NO		
		Regarding the frequency of disinfection, is disinfecting occurring MORE OFTEN, LESS OFTEN, or ABOUT THE SAME as before COVID?		
		With regards to the practice, we would like to ask if there has been a change in the way disinfectants are applied. For each of the following application methods, can you indicate if it has INCREASED, DECREASED, or STAYED ABOUT THE SAME because of COVID? Then terminate survey		Spray
				Wipe
				Mop
				Soak
				Other
10. Is an outside service contracted to disinfect the patient rooms? YES NO	If YES: Skip to question 11			
	If NO: Have the disinfection protocols in the patient rooms changed because of COVID? YES NO	If NO: What class or classes of disinfectant are currently used? Jump to question 11		
		If YES: Has the type of disinfectant used in the patient rooms changed because of COVID? YES NO	If NO: What class or classes of disinfectant are currently used?	
			If YES: What class or classes of disinfectant were used before the pandemic? What class or classes of disinfectant are used now?	
		Has the concentration of disinfectant used changed because of COVID? YES NO		
		Regarding the frequency of disinfection, is disinfecting occurring MORE OFTEN, LESS OFTEN, or ABOUT THE SAME as before COVID?		
		With regards to the patient rooms, we would like to ask if there has been a change in the way disinfectants are applied. For each of the following application methods, can you indicate if it has INCREASED, DECREASED, or STAYED ABOUT THE SAME because of COVID?		Spray
				Wipe
				Mop
				Soak
				Other
11. Is the waiting room open to patients? YES NO	If NO: Skip to question 12			
	If YES: Is an outside service contracted to disinfect the waiting room? YES NO	If YES: jump to question 12		
		If NO: Have the disinfection protocols in the waiting room changed because of COVID? YES NO	If NO: What class or classes of disinfectant are currently used? Jump to question 12	
			If YES: Has the type of disinfectant used in the waiting room changed because of COVID? YES NO	If NO: What class or classes of disinfectant are currently used?
				If YES: What class or classes of disinfectant were used before the pandemic? What class or classes of disinfectant are used now?
			Has the concentration of disinfectant used changed because of COVID? YES NO	
			Regarding the frequency of disinfection, is disinfecting occurring MORE OFTEN, LESS OFTEN, or ABOUT THE SAME as before COVID?	
			With regards to the waiting room, we would like to ask if there has been a change in the way disinfectants are applied. For each of the following application methods, can you indicate if it has INCREASED, DECREASED, or STAYED ABOUT THE SAME because of COVID?	Spray
				Wipe
				Mop
				Soak
				Other
12. Is there a radiology room? YES NO	If NO: Skip to question 13			
	If YES: Is an outside service contracted to disinfect the radiology room? YES NO	If YES: jump to question 13		
		If NO: Have the disinfection protocols in the radiology room changed because of COVID? YES NO	If NO: What class or classes of disinfectant are currently used? Jump to question 13	
			If YES: Has the type of disinfectant used in the radiology room changed because of COVID? YES NO	If NO: What class or classes of disinfectant are currently used?
				If YES: What class or classes of disinfectant were used before the pandemic? What class or classes of disinfectant are used now?
			Has the concentration of disinfectant used changed because of COVID? YES NO	
			Regarding the frequency of disinfection, is disinfecting occurring MORE OFTEN, LESS OFTEN, or ABOUT THE SAME as before COVID?	
			With regards to the radiology room, we would like to ask if there has been a change in the way disinfectants are applied. For each of the following application methods, can you indicate if it has INCREASED, DECREASED, or STAYED ABOUT THE SAME because of COVID?	Spray
				Wipe
				Mop
				Soak
				Other
13. Is there a sterilization room? YES NO	If NO: Skip to question 14			
	If YES: Is an outside service contracted to disinfect the sterilization room? YES NO	If YES: jump to question 14		
		If NO: Have the disinfection protocols in the sterilization room changed because of COVID? YES NO	If NO: What class or classes of disinfectant are currently used? Jump to question 14	
			If YES: Has the type of disinfectant used in the sterilization room changed because of COVID? YES NO	If NO: What class or classes of disinfectant are currently used?
				If YES: What class or classes of disinfectant were used before the pandemic? What class or classes of disinfectant are used now?
			Has the concentration of disinfectant used changed because of COVID? YES NO	
			Regarding the frequency of disinfection, is disinfecting occurring MORE OFTEN, LESS OFTEN, or ABOUT THE SAME as before COVID?	
			With regards to the sterilization room, we would like to ask if there has been a change in the way disinfectants are applied. For each of the following application methods, can you indicate if it has INCREASED, DECREASED, or STAYED ABOUT THE SAME because of COVID?	Spray
				Wipe
				Mop
				Soak
				Other
14. Is there a communal area for patients to wash their mouth, brush teeth, etc.? YES NO	If NO: Skip to question 15			
	If YES: Is an outside service contracted to disinfect the communal area? YES NO	If YES: Jump to question 15		
		If NO: Have the disinfection protocols in the communal area changed because of COVID? YES NO	If NO: What class or classes of disinfectant are currently used? Jump to question 15	
			If YES: Has the type of disinfectant used in the communal area changed because of COVID? YES NO	If NO: What class or classes of disinfectant are currently used?
				If YES: What class or classes of disinfectant were used before the pandemic? What class or classes of disinfectant are used now?
			Has the concentration of disinfectant used changed because of COVID? YES NO	
			Regarding the frequency of disinfection, is disinfecting occurring MORE OFTEN, LESS OFTEN, or ABOUT THE SAME as before COVID?	
			With regards to the communal area, we would like to ask if there has been a change in the way disinfectants are applied. For each of the following application methods, can you indicate if it has INCREASED, DECREASED, or STAYED ABOUT THE SAME because of COVID?	Spray
				Wipe
				Mop
				Soak
				Other
15. Is there an operating room? YES NO	If NO: Skip to question 16			
	If YES: Is an outside service contracted to disinfect the operating room? YES NO	If YES: Jump to question 16		
		If NO: Have the disinfection protocols in the operating room changed because of COVID? YES NO	If NO: What class or classes of disinfectant are currently used? Jump to question 16	
			If YES: Has the type of disinfectant used in the operating room changed because of COVID? YES NO	If NO: What class or classes of disinfectant are currently used?
				If YES: What class or classes of disinfectant were used before the pandemic? What class or classes of disinfectant are used now?
			Has the concentration of disinfectant used changed because of COVID? YES NO	
			Regarding the frequency of disinfection, is disinfecting occurring MORE OFTEN, LESS OFTEN, or ABOUT THE SAME as before COVID?	
			With regards to the operating room, we would like to ask if there has been a change in the way disinfectants are applied. For each of the following application methods, can you indicate if it has INCREASED, DECREASED, or STAYED ABOUT THE SAME because of COVID?	Spray
				Wipe
				Mop
				Soak
				Other
16. Is there a recovery room? YES NO	If NO: Terminate survey			
	If YES: Is an outside service contracted to disinfect the recovery room? YES NO	If YES: Terminate Survey		
		If NO: Have the disinfection protocols in the recovery room changed because of COVID? YES NO	If NO: What class or classes of disinfectant are currently used? Terminate survey	
			If YES: Has the type of disinfectant used in the recovery room changed because of COVID? YES NO	If NO: What class or classes of disinfectant are currently used?
				If YES: What class or classes of disinfectant were used before the pandemic? What class or classes of disinfectant are used now?
			Has the concentration of disinfectant used changed because of COVID? YES NO	
			Regarding the frequency of disinfection, is disinfecting occurring MORE OFTEN, LESS OFTEN, or ABOUT THE SAME as before COVID?	
			With regards to the recovery room, we would like to ask if there has been a change in the way disinfectants are applied. For each of the following application methods, can you indicate if it has INCREASED, DECREASED, or STAYED ABOUT THE SAME because of COVID? Terminate survey	Spray
				Wipe
				Mop
				Soak
				Other

Ethical considerations

The study design, questionnaire survey tool, and phone script were reviewed by Via College of Osteopathic Medicine’s Institutional Review Board (IRB) prior to conducting the study. The IRB determined that because the study was evaluating changes in the medical facility and not employees or patients of the facility, this study did not constitute Human Subjects Research and did not fall under the purview of the IRB. The survey questions and the phone script were specifically crafted to ensure questions focused on the medical facility itself and did not ask about patients and employees, or the viewpoints of patients and employees.

State selection

To collect data that were most representative of the United States, states were ranked according to where they fell compared to the national average for 28 different health metrics covering the time period from 2016 to 2019 as obtained from the Kaiser Family Foundation database (Table 4) [12]. The metrics specifically assessed adults and were not separated by gender or ethnicity. The three states immediately above and below the national average for each of the 28 metrics were identified. The six states that fell most frequently within this range were selected for the study and based on this approach, the states considered most representative of the average conditions in the United States included Arizona, Florida, Illinois, Michigan, Pennsylvania, and Virginia.

**Table 4:** Kaiser Family Foundation (KFF) health metrics This study chose 28 health metrics from the KFF database In the process of state selection. These metrics assessed overall health status, mental health and substance abuse, healthcare costs, and demographics.

HEALTH STATUS METRICS	MENTAL HEALTH AND SUBSTANCE ABUSE METRICS
Adult self-reported health status	Average number of poor mental health days in the last 30 days
Total infant deaths	Individuals reporting alcohol dependence or abuse in the past year
Life expectancy at birth (in years)	Total suicide deaths and age-adjusted suicide rate
Adults who report smoking	Drug overdose death rate (per 100,000 population)
Adults who are obese	
Adults who report participation in any physical activity or exercise	HEALTH COSTS AND BUDGET METRICS
Age-adjusted cancer incidence rate (per 100,000 population)	Health care expenditures per capita
Adults who report ever being told by a doctor that they have diabetes	Private health insurance spending per enrollee
Adults who report being told by a doctor that they have cardiovascular disease	Hospital adjusted expenses per inpatient day
Adults who report being told by a doctor that they have hypertension	
Adults who report being told by a doctor that they have kidney disease	DEMOGRAPHICS AND ECONOMY METRICS
Adults who report currently having asthma	Total number of residents
Adults who report ever being told they have COPD, emphysema, or chronic bronchitis	Distribution of total population by federal poverty level
Influenza and pneumonia deaths per 100,000 population	Median annual household income
Adults who report having arthritis	Distribution of non-elderly population by household employment status
Non-institutionalized population who reported a disability	
Adults who report visiting the dentist or dental clinic within the past year	

Survey administration

Once these six states were identified, lists of hospitals, private practices, and dental offices in each state were created. The American Hospital Directory [13] was utilized to create a list of hospitals, and each state’s Department of Health [14-19] was used to identify dental offices. Private practices were selected from an online COVID-19 Atlas [20] which identified all practices available for management of COVID-19 in the selected states. There was no overlap of specific medical facilities between the three lists. Within the lists, the order of contact was randomized to prevent call bias. Surveys were administered by phone. Respondents were asked if they would prefer to conduct the survey online and if so, were emailed a link to the same survey. All calls and emails, both completed and declined, were recorded in Qualtrics. For all the three surveys, the first question relating to disinfection practices asked whether an outside cleaning agency was used to disinfect the entire facility or a specific area within the facility. When an outside cleaning service was utilized for the entire facility, the survey was terminated to maintain the focus on the medical facility and not the procedures of the cleaning services. This survey result was documented but no data relating to disinfection practices were collected. For facilities that utilized cleaning services for a portion of the practice, data were collected for the areas of the facility that the practice was responsible for, and no disinfection data were collected for regions cleaned by the cleaning service (see Tables 1-3 for the question formats). Data collection continued for two years between January 2021 and 2023. The survey was terminated after two years because a high turn over in practice staff meant that survey respondents may not have been employed pre-pandemic and may not have knowledge about changes in disinfection practices due to the pandemic. Additionally, at the time of survey termination, many of the changes in disinfection protocols had become routine which may have made it difficult for respondents to remember pre-pandemic disinfection protocols.

Most hospitals employed infection control officers who were directly available to answer the survey questions while conversations with private practices and dental clinics were mainly with practice managers or nurses. Many private practices were owned by corporates, so decisions about cleaning methods were handled at the corporate level rather than by specific practices. Even so, the respondents in the practices were able to provide information on changes if any in the product type, frequency of use, and the method of application.

Data analysis process

Data were analyzed to identify any changes in disinfection practices due to the COVID-19 pandemic including changes in the disinfectant product, disinfectant concentration, disinfection frequency, and methods of disinfection. All survey responses were coded in a structured, numerical format to allow for statistical analysis. Unanswered questions were coded as ‘undetermined’ and were not included in the analysis.

## Results

Response data

Over the two-year study period, healthcare facilities were contacted by phone or email. Survey response rates were 11.0%, 3.9%, and 6.1% for hospitals, private practices, and dental practices respectively (Table 5). Though these are seemingly low response rates, recent studies by the Pew Research Center state that the expected survey response rate is around 7.0% in unsolicited telephone surveys and indicates no reduction in its accuracy [21].

**Table 5:** Response by type of healthcare facility The highest response rate was seen among hospitals while private practices had the lowest response rate.

RESPONSE METRIC	HOSPITALS	PRIVATE PRACTICES	DENTAL PRACTICES	OVERALL
Responded	89	56	77	222
Total contacted	812	1427	1259	3498
Response rate	11.0%	3.9%	6.1%	6.3%

Table 6 provides the distribution of the healthcare facilities across urban, suburban, and rural locations as well as the breakdown of practices by general and specialty care. Medical facility types were evenly distributed across rural, suburban, and urban locations. The majority of the private practices and dental clinics that responded provided general care.

**Table 6:** Demographics by location and type of practice The types of medical facilities were relatively evenly distributed across rural, suburban, and urban locations. Most of the private and dental practices surveyed provided general care. The “others" classification includes county government health departments as well as facilities that offer multiple services such as internal medicine, physical therapy, and social work.

DEMOGRAPHICS	HOSPITALS	PRIVATE PRACTICES	DENTAL PRACTICES
Urban	41.8%	33.9%	29.9%
Suburban	26.0%	23.2%	37.7%
Rural	20.0%	42.9%	31.2%
General practice	-	50.0%	62.3%
Specialty practice	-	32.1%	18.2%
Others	-	17.9%	19.5%

Changes in disinfection practices

**Figure 1:**
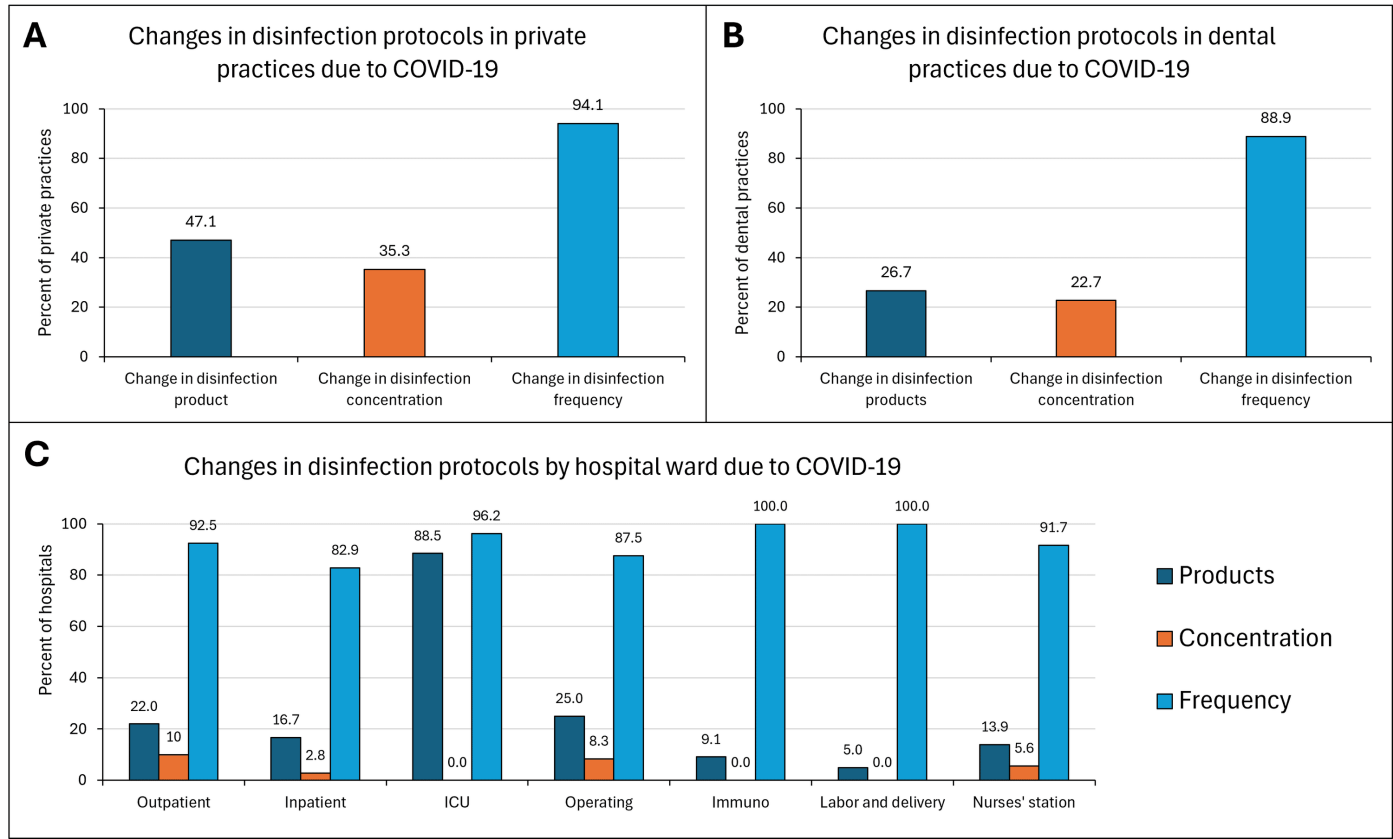
Changes in the disinfection protocols in private practices (A), dental practices (B), and hospitals (C) All types of medical facilities experienced changes in disinfection products and the concentrations of those products. The largest change across healthcare settings was related to the frequency of disinfection.

Private practices, dental practices, and hospitals were asked about the overall change in their disinfection protocols, as well as specific questions pertaining to disinfection products, product concentrations, disinfection methods, and frequency of cleaning in response to the pandemic. Most of the medical facilities surveyed endorsed making changes to their disinfection protocols (Figures 1A-1C). Approximately half of the private practices and a quarter of the dental practices made adjustments to their disinfection products while 35.3% and 22.7% respectively changed the concentration of their disinfectants. In hospitals across all wards, less than 30% made changes in their disinfection products or disinfectant concentration. The notable exception were the ICUs, where 88.5% of hospitals reported an adjustment in the products used for disinfection. This change is not surprising as many ICU wards held critically ill COVID-19 patients and prevention of disease transmission was of paramount importance. The largest change seen in all three types of healthcare facilities was in the disinfection frequency. Over 80% of all the healthcare facilities surveyed reported an increase in the frequency of disinfection.

Changes in the frequency of disinfection

**Figure 2:**
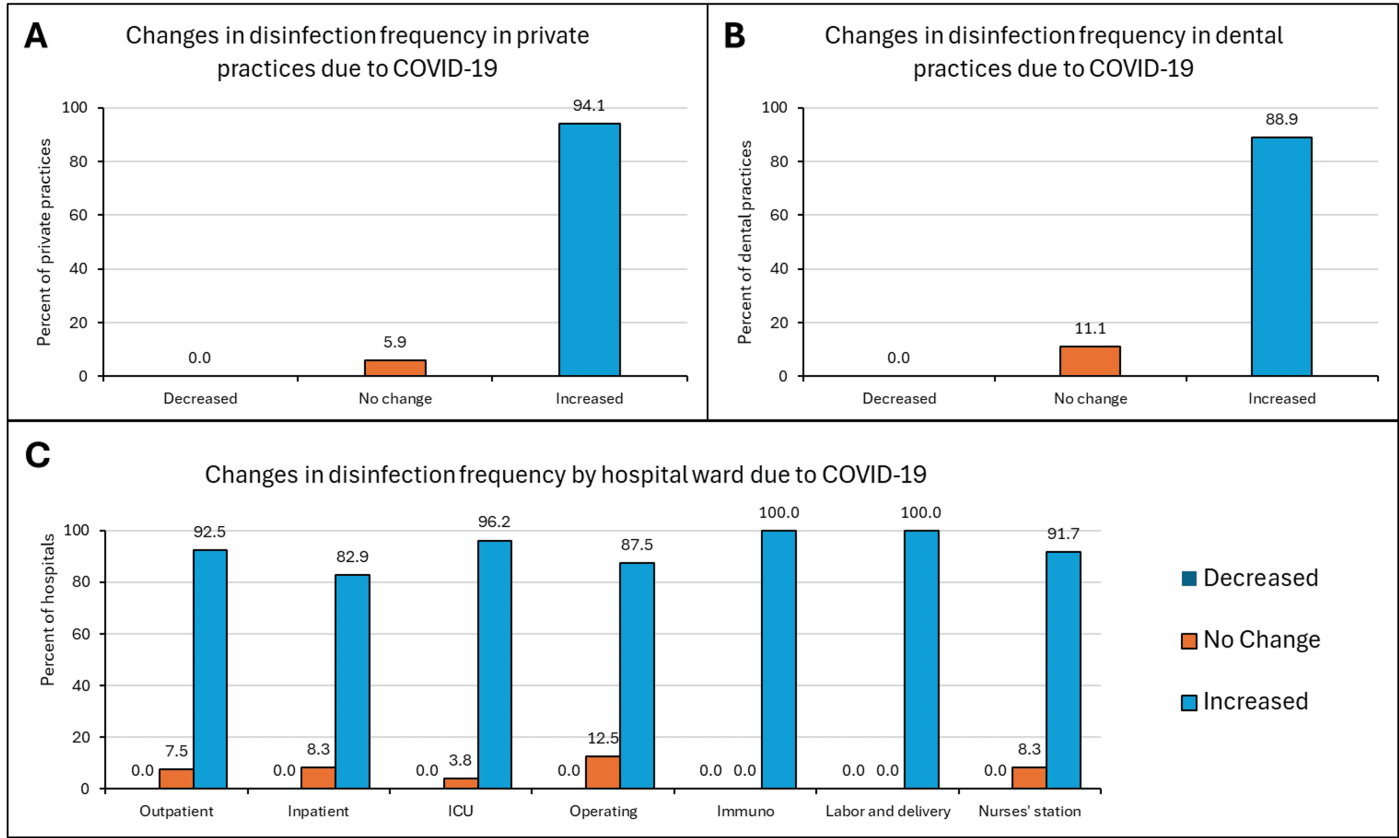
Changes in disinfection frequency in private practices (A), dental practices (B), and hospitals (C) No medical facility decreased the frequency of disinfection while approximately 90% of facilities and hospital wards increased the frequency of disinfection.

A deeper look at the change in the frequency of disinfection showed that 94.1% of the private practices and 88.9% of the dental clinics increased how often they were disinfecting their facilities (Figures 2A-2C). All the hospital wards noted a substantial increase in the frequency of disinfection, with 100% of hospitals with immunocompromised wards and labor and delivery units reporting the greatest increase. No facility reported a decrease in frequency while very few stated they made no changes.

Increase in disinfection methods

**Figure 3:**
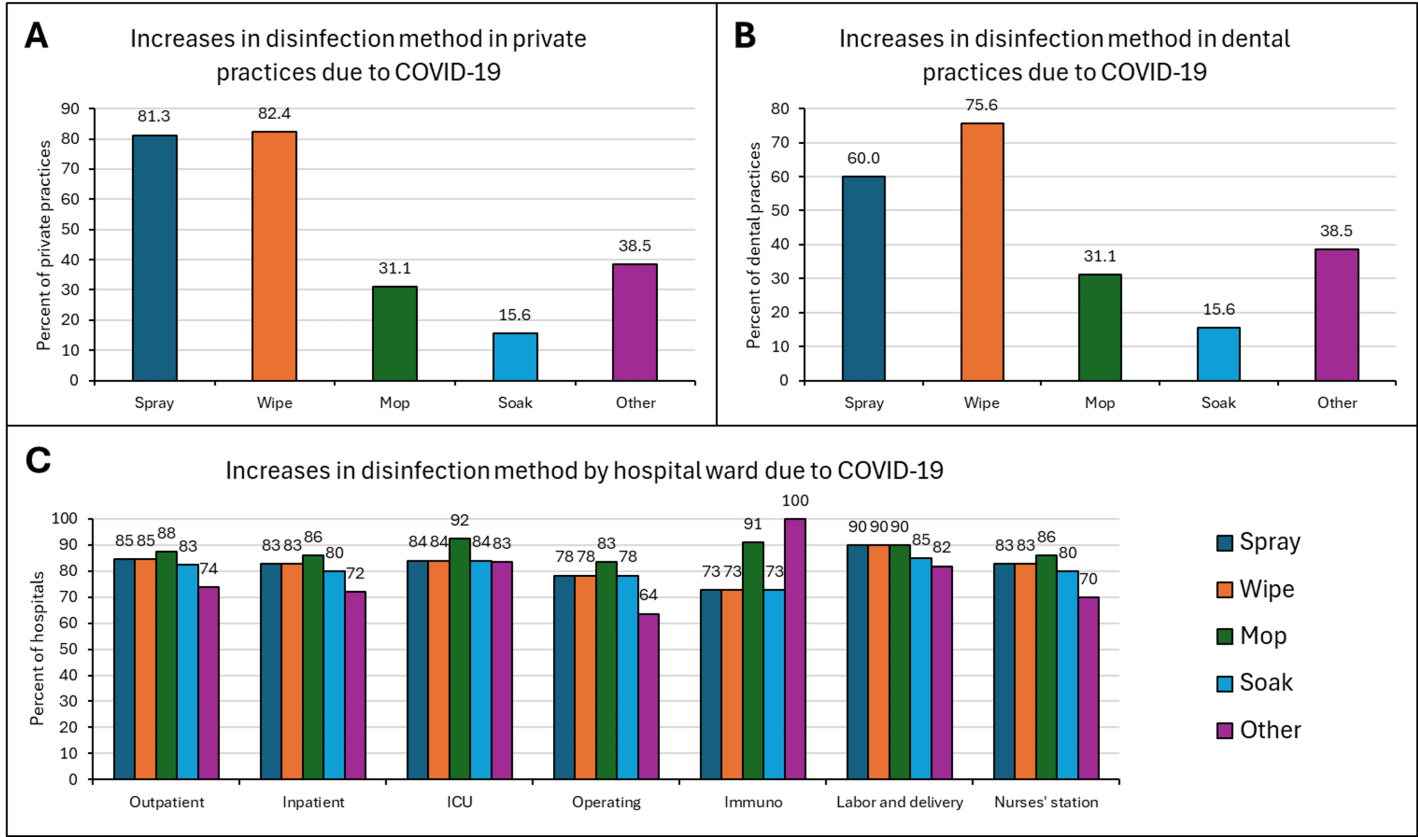
Increases in disinfection method in private practices (A), dental practices (B), and hospitals (C) The greatest increase was seen in wiping with 82.4% of the private practices and 75.6% of the dental practices reporting an increase, followed by spraying, mopping, and soaking. Among hospital wards, an increase in all methods was reported to a similar degree.

The facilities were asked about changes in the way disinfectants were applied, particularly assessing the changes in spraying, wiping, mopping, and soaking. No facility reported a decrease in the application method while very few stated they had made no change. The majority of the private practices and dental clinics increased their wiping and spraying with fewer practices reporting increases in mopping and soaking (Figures 3A-3C). In hospitals, all the application methods were reported as increased to a similar extent in each hospital ward.

Changes in disinfectant class

**Figure 4:**
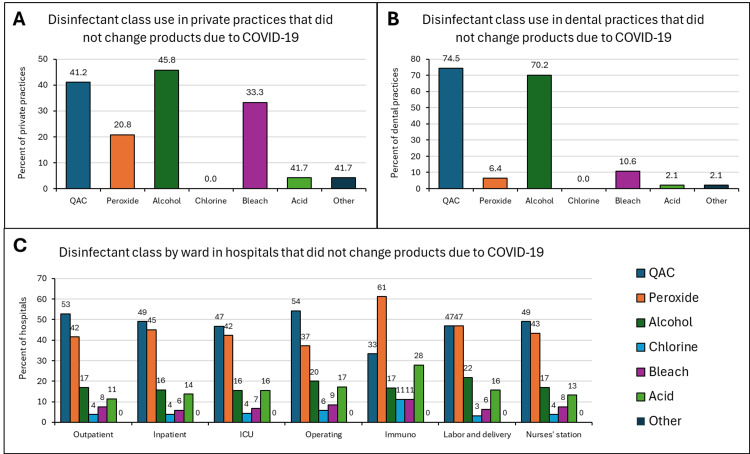
Disinfectant classes used in private practices (A), dental practices (B), and hospitals (C) that did not change products in response to the COVID-19 pandemic Quaternary ammonium compounds (QAC) and alcohol-containing products were the most commonly used classes among this subset of private and dental practices. QAC and peroxide-containing products were used most frequently in all hospital wards. QAC products were used most frequently in all wards except the immunosuppressant ward where peroxide-containing products were used preferentially.

Healthcare facilities were asked which disinfectant products they used before the pandemic and if a change in the product was made in response to the pandemic. If a change in product was made, they were asked which products they now used. In private practices that did not make changes to their disinfectant products, 45.8% and 41.2% of them reported using products containing alcohol and QACs, respectively (Figures 4A-4C). Within the dental practices, 74.5% and 70.2% of the practices continued to use QAC-based and alcohol-based disinfectants, respectively. QAC-containing disinfectants, followed by peroxide-containing products, were the most commonly-used class of disinfectants across hospital wards that did not change their products in response to the COVID-19 pandemic.

**Figure 5:**
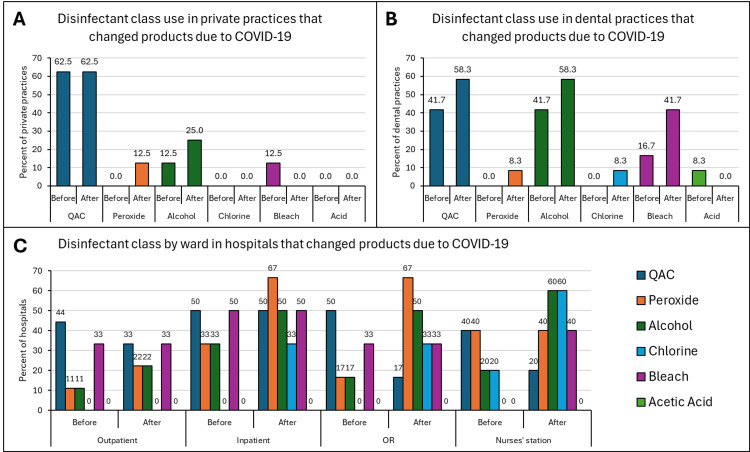
Disinfectant classes used in private practices (A), dental practices (B), and hospitals (C) that did change products in response to the COVID-19 pandemic QAC-based products were among the most used products in private practices, dental clinics, and hospital wards before the pandemic. The proportion of private practices using QACs remained unchanged, but a greater percentage of dental clinics reported using QAC products after the pandemic. QACs continued to be used in hospital wards while an increase in other disinfectant classes like peroxides was seen in outpatient and inpatient wards, and operating rooms. QAC: Quaternary ammonium compound

In private practices that reported a change in their cleaning products in response to the pandemic, there was no change in QAC-based products, with 62.5% of practices reporting use before and after the start of the pandemic. However, 12.5% and 25% of private practices reported switching to disinfectants containing peroxide and alcohol, respectively (Figures 5A-5C). An equal number of dental practices used QAC-based and alcohol-based products before the pandemic (41.7%). The percentage of dental clinics using these two products increased to 58.3% during the pandemic. The most used product type within the hospitals before the pandemic was QAC-based. In response to the pandemic, QACs were still used but to a slightly lesser degree in the outpatient, operating room, and nurses’ station settings.

## Discussion

Private practices, dental clinics, and hospitals all demonstrated increases in the frequency of disinfection and changes to their cleaning methods. Across all healthcare facilities that were surveyed, QAC-containing disinfectants were the first or the second most commonly used products during the COVID-19 pandemic. In some instances, medical facilities switched to using more QAC-based disinfectants.

Ultimately, the predominant use of products containing QACs and the increase in cleaning frequency suggest that QAC exposure increased during the pandemic. There is currently a paucity of data documenting both the prevalence and extent of QAC exposure in healthcare settings and the risks this exposure may pose to healthcare workers. Our study shows that there was a nationwide increase in the use of QACs in response to the COVID-19 pandemic. Based on this finding, it could be inferred that healthcare workers in certain settings experienced higher exposure to QACs through increased physical contact and inhalation of these chemicals during the pandemic. However, as our study focused on disinfectant use in various healthcare settings, human exposure was not monitored. Further research needs to be conducted to document healthcare workers' exposure and assess the risk of the harmful effects associated with these disinfectants.

A number of studies have detected QACs in human blood [6,22,23]. It is unclear whether QACs truly accumulate in the human blood over time. One study analyzed human blood samples before and during the pandemic, determining that QAC concentrations were significantly higher in the latter period [22]. Among the 222 blood samples collected, the median concentration of QACs was 3.41 ng/mL before the pandemic and 6.04 ng/mL during the height of the pandemic [22]. This finding could have been associated with an increased use of QAC-based disinfectants during the pandemic, but no data were collected about concurrent disinfection practices and an association could not be made. A second study showed similar findings, with 80% of blood samples showing quantifiable levels of QACs [6]. However, this study was not designed to identify the time of exposure; therefore, no such conclusion could be drawn. Because these studies all measured blood contamination at a single point in time, no inference can be made regarding the timing and source of exposure. Therefore, it is unclear if QACs can accumulate in blood or other human tissues.

QACs have recently been detected in human breast milk too [24]. Higher QAC levels were present in the breast milk of women who used QAC-containing products more often and disinfected more frequently [24]. Of note, the QAC concentration in breast milk was two times higher in mothers who used QAC-based disinfectant sprays compared to those who used QAC-based wipes [24]. From this study alone, it is not clear whether QACs bioaccumulate at a higher concentration in breast milk than plasma. Similarly, no inference can be made regarding the timing of exposure and the first detection of QACs in breast milk. Answers to these questions could help prevent unintentional exposure of infants and neonates.

While a comparison between human and animal studies needs to be done cautiously, the latter are the foundation of toxicity testing and risk assessment. Risk assessments are evaluations conducted by the EPA to identify whether a chemical poses a risk to the human population. The determination is based on the outcomes of standardized regulatory tests conducted in rodents. These results are then translated to the human population and the risk of exposure is assessed. Risk assessments have been conducted for two of the most common QACs and both were identified as possessing low risk of harm to people [25,26]. However, since then, numerous studies have been published identifying the adverse health effects associated with QAC exposure in both people and animals [2]. There are many reasons why the risk assessments may not have captured negative health outcomes. A main factor could be that they evaluated exposure to one QAC at a time and people are normally exposed to a mixture of QACs. Exposure to mixtures of chemicals can have widely divergent consequences than exposure to a single chemical. Additionally, risk assessments evaluate prescribed endpoints and do not assess many of the negative outcomes identified from QAC exposure. Lastly, it was assumed that ingested QACs were not absorbed into the body but rather passed through the intestines without resulting in systemic exposure. It is now known that ingested QACs are readily absorbed into the body from the gastrointestinal tract, and that the transfer of the disinfectants from surfaces to the hands and then to the mouth is likely the main route of exposure in humans [2]. This means that human exposure was likely underestimated in the risk assessments leading to a lower risk rating.

There are few studies documenting the impact on human health beyond the robust literature reviewed by Arnold et al. (2023) identifying asthma, allergy, dermal, and corneal inflammation and sensitization [2]. One study evaluating the systemic effects linked to QAC exposure on humans found a dose-dependent association between blood QAC levels and the presence of inflammatory cytokines, diminished mitochondrial function, and disruption of cholesterol homeostasis [6]. This study demonstrated direct translation of results from numerous animal studies to the human population giving weight to the concern that QACs may be harmful to humans.

One concerning area of study is that QACs may act as developmental neurotoxicants. QACs cause neural tube birth defects in mice [7]. While it is not known if QAC exposure is linked to altered neural development in humans; it is well documented that teratogenic agents that cause neural tube birth defects in humans also cause neurobehavioral disorders [7]. From rodent studies, it is known that QACs disrupt lipid biosynthesis which is crucial for neurodevelopment [27]. Lipid metabolism is an important process in the proliferation of neural stem progenitor cells and serves as a vital component of the membrane structure [27]. QACs are also potently and selectively toxic to developing oligodendrocytes which are critical for neuronal function [28]. These findings are particularly concerning as QACs readily enter the neonatal mouse brain by crossing the blood-placental and the blood-brain barriers resulting in disruption to the signaling pathways necessary for proper neurodevelopment [27].

One such signaling process is the glutamate receptor signaling pathway, which regulates neuronal migration, synaptogenesis, and differentiation [29]. With QACs exhibiting the ability to impair cholesterol biosynthesis, it is assumed that by entering the neonatal mouse brain, they can also contribute to dysregulation of the glutamate receptor signaling pathway, causing further neurodevelopmental problems [27]. The animal study mentioned above shows the negative effects QACs can have on the developing brain of mice. If this were to be the case in humans, the increased QAC exposure during the pandemic is concerning, particularly for pregnant healthcare workers spending time in these settings.

Another major area of concern is the association between QACs and infertility. Infertility is a significant clinical problem, affecting 8-12% of couples worldwide. This issue is further emphasized by the number of infants born using assisted reproductive technologies (from 61,556 in 2010 to 83,946 in 2019) [30]. While human studies have not yet been conducted, animal studies have identified decreased reproductive function linked to QAC exposure [8]. Mice that were exposed for six months displayed increased time to delivery, longer time in between pregnancies, less mice per delivery, and fewer pregnancies [8]. Another study assessed sex-specific reproductive function impairments in mice. When exposed to QAC-containing products, males showed a decline in sperm motility and concentration, and female mice showed decreased ovulation, fertilization, and implantation as well as an increase in pregnancy loss [3]. Further studies are necessary to establish an association between QACs and reproductive impairment in humans.

Limitations and future studies

Data collected from hospitals were more complete because those facilities had infection control officers who were able to answer questions and most hospitals were able to make their own decisions in terms of disinfection protocols. Since the same was probably not true for a similar proportion of private practices and dental offices, there was unreported information for certain questions. Some private practices and dental practices were owned by corporates and the decisions regarding disinfection practices were made at the corporate level. This may have introduced bias in the survey responses. This was minimized by having practice staff working in the facility most familiar with the procedures utilized there complete the survey, and not the staff at corporate headquarters. Additionally, no questions regarding the rationale for disinfectant selection and procedures were asked. Lastly, while the states selected for this study were chosen to reflect the average states in the United States, only six states were surveyed. Hence, the results may not reflect those of the country as a whole.

Further studies should center around healthcare personnel who worked during the pandemic to determine the long term health effects of the increased exposure. These individuals can aid in establishing or solidifying the harmful role QACs have in human infertility, breastfeeding, and neurodevelopment. Studies are also needed to determine the kinetics of QAC exposure to identify how they are absorbed and how long they remain in the body. This would help determine if they bioaccumulate. Lastly, studies are needed to identify the major routes of exposure for healthcare workers so systemic exposure can be mitigated.

## Conclusions

A review of the data collected in this study shows that the COVID-19 pandemic brought about changes in disinfection practices within hospitals, private practices, and dental clinics. This study helped determine that these healthcare facilities have increased the frequency and methods of disinfection with most of them using QAC-based disinfectants during the COVID-19 outbreak. The reliance on QAC-based products within healthcare settings is an important finding as it indicates heightened exposure to these potentially harmful chemicals with increased possibility for adverse health effects in humans.
