# Correction: Impact of the COVID-19 Pandemic on the Use of Quaternary Ammonium Compound Disinfectants in Healthcare Facilities Within Selected States in the United States of America

**DOI:** 10.7759/cureus.c266

**Published:** 2025-08-26

**Authors:** Callie Moran, Kathrine Gaiko, Tillman Landers, Hannah L Paros, Chang Xu, Elizabeth Fernandez, Brooke Burwell, Tyler Steve, Sasha Margob, Marisela Davis, Karen Price, Madeleine E Chew, Sara Worrill, Brianna R Childress, Sara Safford, Janvi Patel, Jessica Marino, Ashley Azmoodeh, Theresa J McCann, Terry Hrubec

**Affiliations:** 1 Epidemiology and Public Health, Edward Via College of Osteopathic Medicine - Virginia, Blacksburg, USA; 2 Epidemiology and Public Health, Edward Via College of Osteopathic Medicine - Auburn, Auburn, USA; 3 Epidemiology and Public Health, Edward Via College of Osteopathic Medicine - Carolinas, Spartanburg, USA; 4 Preventive Medicine, Epidemiology, Biostatistics, Public Health, Edward Via College of Osteopathic Medicine - Virginia, Blacksburg, USA; 5 Anatomy/Embryology, Edward Via College of Osteopathic Medicine - Virginia, Blacksburg, USA

The Ethics Statement and Conflict of Interest Disclosures section of this article has been corrected to indicate that this study did involve human subjects or tissue.

